# The Role of Noise in Specific Detectivity of InAs/GaSb Superlattice MWIR Bariodes

**DOI:** 10.3390/s21217005

**Published:** 2021-10-22

**Authors:** Krzysztof Czuba, Łukasz Ciura, Iwona Sankowska, Ewa Papis-Polakowska, Agata Jasik

**Affiliations:** 1 Łukasiewicz Research Network—Institute of Microelectronics and Photonics, al. Lotników 32/46, 02-668 Warsaw, Poland; krzysztof.czuba@imif.lukasiewicz.gov.pl (K.C.); iwona.sankowska@imif.lukasiewicz.gov.pl (I.S.); ewa.papis.polakowska@imif.lukasiewicz.gov.pl (E.P.-P.); 2 Department of Electronics Fundamentals, Rzeszow University of Technology, W. Pola 2, 35-959 Rzeszow, Poland; lciura@prz.edu.pl

**Keywords:** infrared sensors, InAs/GaSb superlattice, barrier photodiode, noise performance, specific detectivity, low-frequency noise

## Abstract

In this paper, the results of the electrical, noise, and optical characterization of p-i-n and p-B-i-n diodes with AlSb and 4 ML AlSb/8 ML GaSb superlattice barriers in High-Operating Temperature conditions, are presented. Experimental and theoretical noise parameters were compared. Both dark current and noise analysis showed that the p-B_p_bulk_-i-n bariode had the best performance. P-i-n photodiodes had the highest experimental value of specific detectivity (D*) of 6.16 × 10^9^ Jones at 210 K and zero bias. At about −1 V reverse bias, the bariode with AlSb/GaSb electron barrier caught up to it and both devices achieved D* = (1–1.1) × 10^8^ Jones. Further optimization of the superlattice-based electron barrier should result in the improvement of bariode performance at a smaller bias, at which better noise performance is more pronounced. It was shown that neglecting the low-frequency noise component can lead to a significant overestimation of detectivity. The simple method of incorporation of low-frequency noise contribution in the detectivity calculation, without time-consuming measurements, has been proposed.

## 1. Introduction

Infrared photodetectors are widely used in many branches of modern society such as environment monitoring, imaging, medicine, spectroscopy, or the military. Presently, mercury-cadmium-telluride-based technology dominates the market. However, national and international legislation works to eliminate heavy metal usage in the production of consumer products, including Hg and Cd. As a result, research to find better and safer materials is undergoing in facilities around the world. At present, two of the best candidates are antimonide-based type-II superlattices (SL) InAs/GaSb and InAs/InAsSb [1,2,3,4,5]. They allow for the utilization of bandgap engineering design structures with long-wavelength absorption edge practically in the entire mid-infrared spectral range [6]. Furthermore, both their growth and processing technology matured in the last decade to the point, at which discrete photodetectors, as well as focal plane arrays based on them, are becoming more common [7,8,9].

Lately, more and more attention is paid to the High-Operating Temperature (HOT) photodetectors, which work at temperatures from 200 K to 300 K and beyond [10,11,12]. In such devices, low temperatures are achieved through the use of thermoelectric cooling. They have many advantages over cryogenically cooled detectors. In particular, they allow for further decreasing the size and weight, lowering production costs as well as better power usage efficiency. As a result, their applications can be more versatile and more easily spread into the consumer market.

Increasing the operating temperature of SL-based infrared photodetectors can be achieved using different approaches. One is to use novel architecture, such as in Interband Cascade Detectors or Quantum Cascade Detectors [13,14]. Presently, the main disadvantage of such structures is their complexity. They require considerable computational effort during the design and optimization stages. Furthermore, they are hard to realize epitaxially, as the margin of error required for their proper operation is very small. Another approach is to introduce unipolar carrier barriers into mature detector designs (e.g., photoconductor or p-i-n photodiode), the purpose of which is the suppression of dark current and noise without negatively influencing the photocurrent [15,16,17]. The barriers can be placed for one or two types of carriers, depending on the detector structure. They can be made of either bulk material (e.g., AlSb, AlAsSb, AlGaSb) or superlattice (e.g., InAs/AlSb, AlSb/GaSb). The latter allows for better optimization of the photodetector band structure due to the utilization of bandgap engineering.

This paper is focused on photodiodes with (p-B_p_-i-n) and without (p-i-n) electron barriers, based on a double heterojunction design. The B_p_ in p-B_p_-i-n pertains to a p-type electron barrier made of either AlSb or AlSb/GaSb superlattice. The analysis of dark current, noise, and optical response is presented. The emphasis was put on the role of noise in the performance of these infrared photodetectors. Furthermore, a comparison between two semi-empirical approximations (the first including thermal and shot noise, and the second including thermal, shot, and low-frequency noise) and actual measurements is presented. The issues arising from the omission of low-frequency noise during the calculation of specific detectivity and resulting errors are addressed. Usually, noise figures for InAs/GaSb-superlattice-based devices are given for low temperatures <150 K, which is understandable since cryogenically-cooled detectors are most commonly used [18,19,20]. In this paper, all noise characteristics are shown in the HOT temperature range in addition to the low-temperature characterization. Furthermore, to the best of our knowledge, there are no low-frequency noise measurements reported for bariodes on GaAs substrate, in contrast to recently reported results for various detectors on GaSb substrate [19,21,22].

## 2. Fabrication of InAs/GaSb Superlattice Photodetectors

**Figure 1:**
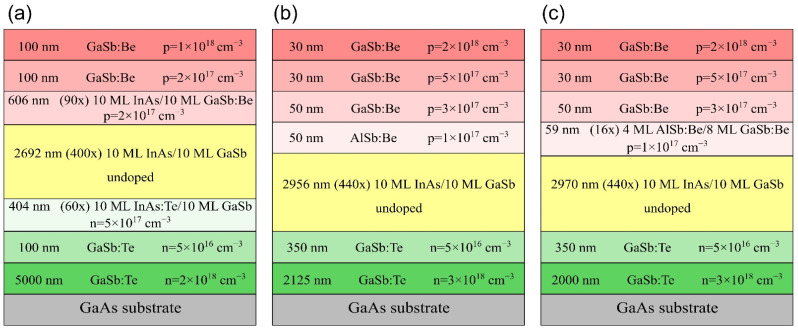
Schematic diagrams of photodiodes: (**a**) p-i-n, (**b**) p-B_p_bulk_-i-n with AlSb barrier, and (**c**) p-B_p_SL_-i-n with 4 ML AlSb/8 ML GaSb superlattice barrier.

In this work, three types of photodiodes were compared, namely p-i-n, p-B_p_bulk_-i-n with AlSb electron barrier, and p-B_p_SL_-i-n with type-I AlSb/GaSb superlattice electron barrier. In Figure 1, schematic diagrams of each photodetector are shown. All samples were deposited on (100) GaAs substrates.

**Figure 2:**
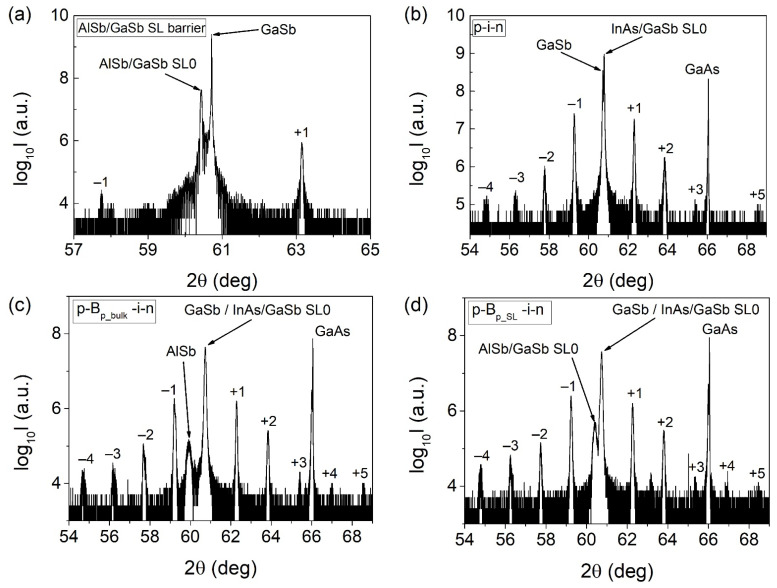
Measured 2θ/ω curves for (**a**) 4 ML AlSb/8 ML GaSb barrier superlattice and photodiodes: (**b**) p-i-n, (**c**) p-B_p_bulk_-i-n with AlSb barrier, (**d**) p-B_p_SL_-i-n with 4 ML AlSb/8 ML GaSb superlattice barrier.

Photodetector heterostructures were grown using molecular beam epitaxy in Riber 32P reactor. In both types of structures, p-type and n-type contact layers were made of highly doped (~10^18^ cm^−3^) GaSb material. The absorption region consisted of 400 to 440 periods of type-II 10 ML InAs/10 ML GaSb superlattice with InSb-like and GaAs-like interfaces. The epitaxial growth of the superlattice was described elsewhere [23]. Before fabrication of p-B_p_SL_-i-n photodiodes, the growth of 4 ML AlSb/8 ML GaSb superlattice was optimized. A series of test processes were performed, which culminated in high-quality material. In Figure 2a, the 2θ/ω curve measured for AlSb/GaSb superlattice using the high-resolution x-ray diffraction (HRXRD) method is shown. From its analysis, the following information was obtained: AlSb layer thickness—4.1 ML (12.5 Å; ML stands for monolayer), GaSb layer thickness—8.3 ML (25.3 Å), and perpendicular lattice mismatch (Δa/a)_⊥_ of 4240 ppm. The latter is defined as follows:

(1) $${\left(\Delta a/a\right)}_{⊥}=\frac{a_{⊥\_\mathrm{SL}0}-{\;a}_{⊥\_\mathrm{GaSb}}}{a_{⊥\_\mathrm{GaSb}}},$$

where a_⊥_SL0_ is the perpendicular lattice constant of the superlattice, for which lattice mismatch is calculated, and a_⊥_GaSb_ is the perpendicular lattice constant for GaSb substrate (for test AlSb/GaSb superlattice) or GaSb buffer layer (for photodiode/bariode structures). The zeroth-order satellite peak has an FWHM of 144 arcsec. The presence of Pendellösung peaks confirmed parallelism of crystallographic planes. Furthermore, this AlSb/GaSb superlattice exhibited a smaller lattice-mismatch to the GaSb than bulk AlSb.

Device heterostructures underwent structural quality tests using the HRXRD method. In Figure 2b–d, 2θ/ω curves are shown. The summary of structural details of photodiode heterostructures under consideration and lattice mismatch (Δa/a)_⊥_ between both absorber superlattices and barriers and GaSb buffer are given in Table 1. For all samples, higher-order satellite peaks up to the fifth order are observable, which is an indication of their high quality. The AlSb/GaSb superlattice electron barrier in p-B_p_SL_-i-n heterostructure differed slightly from the test one. The layer thicknesses were AlSb—4.4 ML, GaSb—7.6 ML, and (Δa/a)_⊥_ was 4950 ppm.

Photodetectors were designed to operate in back-side illumination configuration and p-type contact layers are on the top of the structure. There were three reasons behind this choice. Firstly, it was to minimize losses related to the high free-carrier absorption in p-type GaSb in the mid-wavelength spectral range. Secondly, materials used as electron barriers have a large value of (Δa/a)_⊥_ to the buffer, namely 11,800 ppm for AlSb and 4950 ppm for 4 ML AlSb/8 ML GaSb. It could result in worsening of both structural and optical quality of 10 ML InAs/10 ML GaSb absorber superlattice if grown on top of such barrier layers. It would also negatively impact figures of merit of the photodetector. Finally, a double pass of infrared light through the absorption region could be achieved in such a configuration due to reflection from the top contact. Barriers were intentionally doped with beryllium to minimize the effects of valence band offsets between them and neighboring layers.

After the growth, heterostructures were further processed. Circular mesa structures were formed using the reactive ion etching-inductively coupled plasma dry etching method. The diameters of the photodiodes were 300 μm. Electrical contacts were made by the deposition of Ti/Au metallization using the magnetron sputtering method. After dicing, sample photodetectors were mounted onto test sub-assemblies using wire bonding. Finally, dark current, noise, and optical characterizations were performed.

## 3. Results and Discussion

### 3.1. Dark Current Characteristics

**Figure 3:**
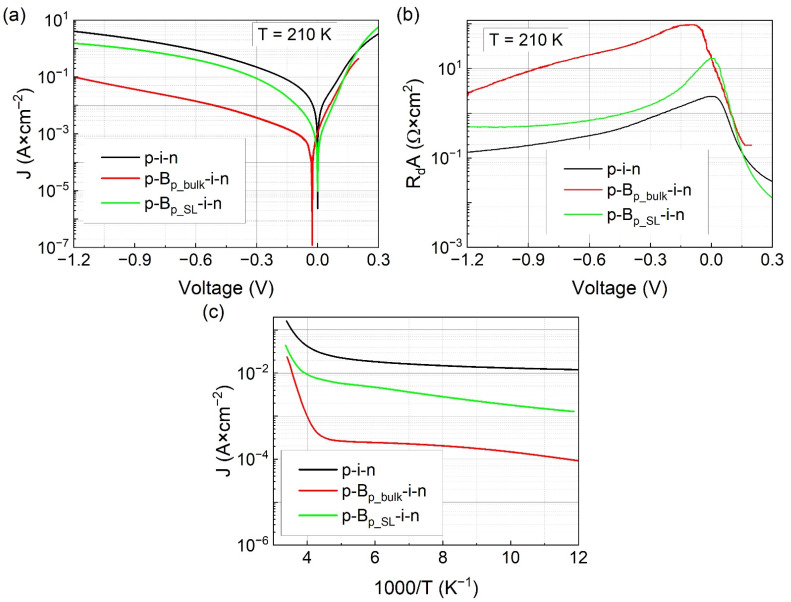
(**a**) Dark current density versus voltage and (**b**) differential resistance and area product versus voltage for p-i-n, p-B_p_bulk_-i-n, and p-B_p_SL_-i-n photodiodes at 210 K. (**c**) Dark current density versus 1000/T for all photodiodes at a reverse bias of 50 mV.

Dark current-voltage characteristics were measured for each photodiode in a wide range of temperatures using a setup consisting of Keithley 2612A source-measure instrument and Janis CCS-150 closed-cycle cryocooler. Samples were isolated from the environmental background radiation using a cold shield, which was cooled to around 10 K during the measurements. Differential-resistance-area product (R_d_A) as a function of bias was calculated from experimental I–V data. In Figure 3a,b, J–V and R_d_A–V curves for considered photodiodes at 210 K are shown, respectively. This temperature was chosen as it corresponds to the one achievable through miniature three-stage thermoelectric coolers. Large differences in electrical characteristics between particular photodetectors were observed. The lowest dark current density was obtained for the p-B_p_bulk_-i-n bariode, whereas the highest was for the p-i-n diode. Comparison between bariodes indicated that p-B_p_SL_-i-n has a higher dark current. Both unbiased barrier photodiodes had similar values of R_0_A parameter of about 17 Ωcm^2^ for p-B_p_bulk_-i-n and ~18 Ωcm^2^ for p-B_p_SL_-i-n. However, for the latter device, it was the highest value whereas p-B_p_bulk_-i-n achieved a higher value of ~100 Ωcm^2^ with a small reverse bias of 104 mV. The classic p-i-n photodiode had a maximum R_d_A value of 2.4 Ωcm^2^ for zero bias. It is noteworthy that the photodetectors in this paper were not passivated, which could further decrease the dark current density.

In Figure 3c, dark current densities at −50 mV bias are plotted as a function of reciprocal temperature. The p-i-n photodiode had the highest dark current density in the entire temperature range of 77 K–300 K. The p-B_p_bulk_-i-n bariode had the smallest dark current density and outperformed the p-B_p_SL_-i-n by more than one order of magnitude for T < 200 K. In Table 2, activation energies E_a2_ and E_a3_ obtained from the analysis of the curves in Figure 3c and their corresponding temperature ranges are given. All photodiodes had very small (<10 meV) activation energies in low-temperature ranges (ΔT_2_ and ΔT_3_), which suggests surface leakage channel as the main shunt current source. A more detailed analysis is needed to identify a specific mechanism. The behavior of photodiodes differs more at higher temperatures (ΔT_1_). The activation energies E_a1_ for the p-B_p_SL_-i-n bariode and the p-i-n photodiode were close to half of their bandgap energy at 300 K (E_g_300 K_), which were equal to ≈250 meV and 235 meV, respectively. These values indicate that the operation of these detectors in the ΔT_1_ temperature range is limited by the generation-recombination (g-r) component of the dark current and/or shunt currents since it was shown that they can exhibit similar behavior and are influenced by the defect states in the junction area [24]. On the other hand, p-B_p_bulk_-i-n had the highest activation energy, which was closer to the value of E_g_300 K_ ≈ 250 meV (E_a1_ ≈ 0.88 × E_g_300 K_). This suggests that both diffusion and g-r dark current components are limiting its performance, with the former being the dominant one. The values of E_g_300 K_ for p-i-n photodiode were estimated using the Varshni relation, which is described in Section 3.3. On the other hand, the E_g_300 K_ for both bariodes was determined from measurements of spectral current responsivity R_I_ at 300 K (not shown in the paper).

### 3.2. Noise Characteristics

**Figure 4:**
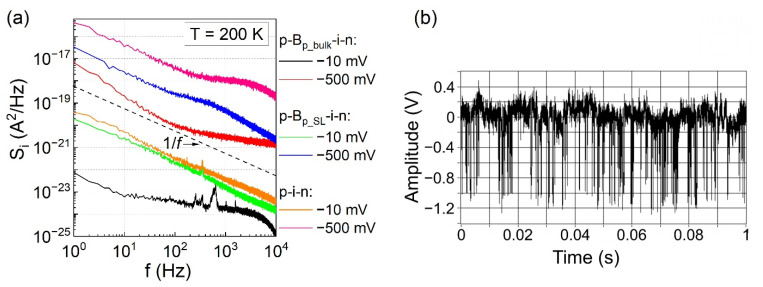
(**a**) The noise power spectral density versus frequency for considered photodiodes, measured at 200 K for two bias points. (**b**) Noise signal in the time domain for p-B_p_bulk_-i-n structure recorded at the output of transimpedance amplifier (k_iu_ = 10^8^ V/A), at T = 200 K and U = −50 mV.

The setup used for low-frequency noise measurements and the small-signal equivalent circuit of a photodiode were described in Ref. [25]. In Figure 4a, power spectral densities measured at T = 200 K for the low and high bias voltages are shown. In general, power spectral densities exhibit 1/f dependence, but some Lorentzian (S_i_ ~1/(1 + (f/const.)^2^) inclusions appear, especially for the low biased detector with a bulk AlSb barrier. The analysis of the noise signal in the time domain, illustrated in Figure 4b, reveals that Lorentzian is connected with random telegraph noise (RTN). Such noise is attributed to the carrier capture/emission by extended defects present in the depletion region [26]. At a small voltage bias of −10 mV, the low-frequency noise magnitude is the lowest for the p-B_p_bulk_-i-n detector. The significant difference in the magnitude is especially observed for low bias voltage and at low frequencies. For U = −500 mV, p-i-n photodiode had the highest noise magnitude in the entire measured frequency range, whereas p-B_p_bulk_-i-n had the smallest. The p-B_p_SL_-i-n was in-between, however, its S_i_ decreased to the level of p-B_p_bulk_-i-n at f ≈ 10 kHz.

**Figure 5:**
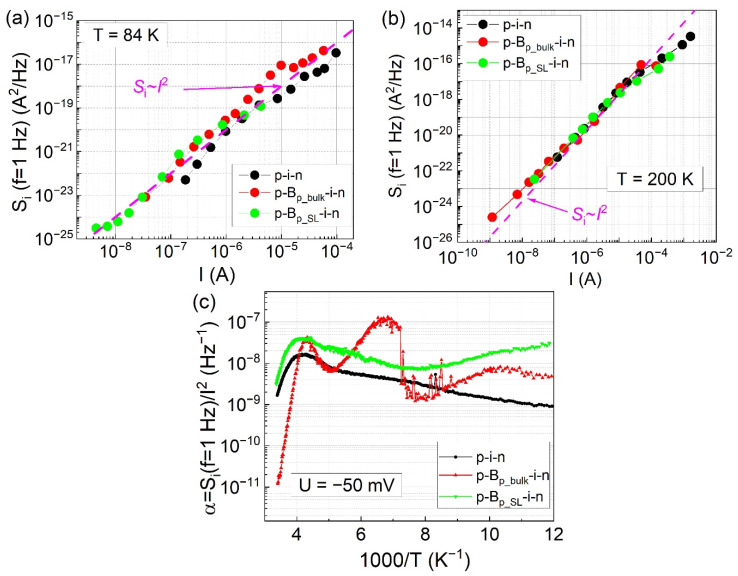
S_i_(I) characteristics measured at (**a**) T = 84 K and (**b**) T = 200 K. (**c**) The noise coefficient α, obtained for noise measurements at voltage bias U = −50 mV, as a function of reciprocal temperature for considered photodiodes.

In Figure 5a,b, power spectral density magnitude S_i_(f = 1 Hz) measured at T = 84 K and T = 200 K are shown as a function of bias current I. The dashed line in this figure follows dependence S_i_(f = 1 Hz) ~I^2^. The measured characteristics S_i_(I) match S_i_(I) ~I^2^ dependence closely. There is no significant difference in the S_i_(I) magnitude for three considered types of photodetectors. If squared, dependence between S_i_(I) and I holds, the noise coefficient can be defined as α = S_i_(f = 1 Hz)/I^2^. The noise coefficients as a function of temperature are shown in Figure 5b. In this experiment, the noise was measured at voltage bias U = −50 mV. In the low-temperature region (T ≈ 84 K), the noise coefficient for all three structures is within the range of 1 × 10^−9^–2 × 10^−8^ Hz^−1^. The current-temperature characteristics (see Figure 3c) show that in the low-temperature region, the leakage shunt current I_sh_ dominates. Different values of noise coefficient α_sh_ = S_i_(f = 1 Hz)/(I_sh_)^2^, related to the shunt current, have been reported in the literature. For InAs/GaSb SL devices, α_sh_ is within the range of 3 × 10^−10^–6 × 10^−6^ Hz^−1^ [21,27]. Our values of α_sh_ lie in the middle of that range and change with the temperature only slightly. In the high-temperature region, the diffusion and generation-recombination currents dominate the total current. It was shown that such current, especially the diffusion component, has a much lower noise coefficient [18,21,27,28,29]. For detectors with InAs/GaSb SL absorber, α_g-r_ = 4.8 × 10^−9^ Hz^−1^ and α_diff_ = 1.9 × 10^−10^ Hz^−1^ were found for generation-recombination and diffusion current, respectively [19]. In the high-temperature region, the noise coefficient decreases slightly for p-i-n, p-B_p_SL_-i-n because the shunt current is still significant for these structures as compared to the diffusion current. For p-B_p_bulk_-i-n, with low shunt current, the noise coefficient drops suggestively reaching a value of 1 × 10^−11^ Hz^−1^, which is much lower than the best (lowest) results found by us in the literature [19]. This translates into good noise performance of the p-B_p_bulk_-i-n detector at temperatures, where current is limited by its diffusion component.

### 3.3. Optical Characteristics

Spectral current responsivity measurements were performed in the range of 3–6 μm at 210 K. The experimental setup consisted of Horiba MicroHR motorized monochromator equipped with SiC-based IR source and ruled grating with blaze wavelength of 5000 nm. The optical signal was mechanically chopped and detected using the Stanford Research Systems SR830 DSP lock-in amplifier. A calibrated lithium tantalate pyroelectric detector with a BaF_2_ window was used to measure the reference signal. Sample photodetectors were placed in Janis CCS-150K closed-cycle cryocooler with a KBr window.

**Figure 6:**
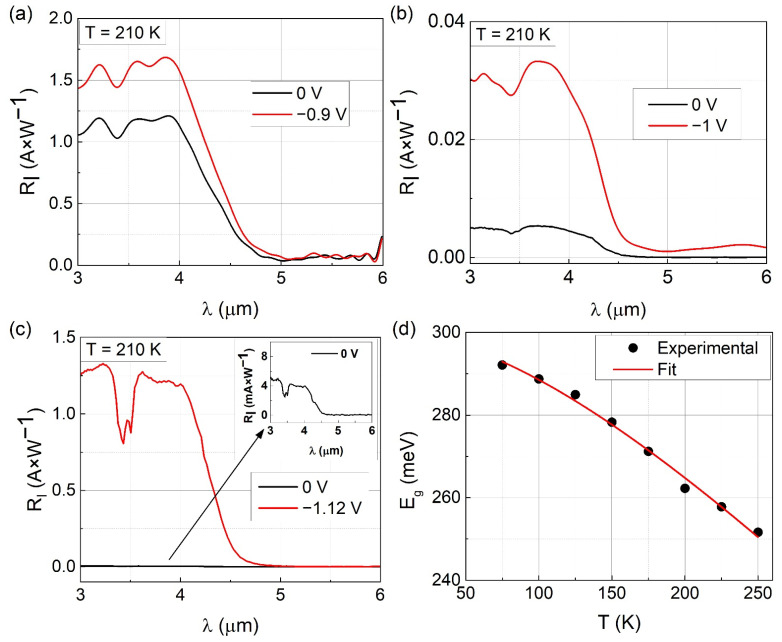
Spectral current responsivity (R_I_) for (**a**) p-i-n, (**b**) p-B_p_bulk_-i-n, and (**c**) p-B_p_SL_-i-n photodiodes at 210 K, at two biases. (**d**) Bandgap energy of p-i-n photodiode absorber region at various temperatures and fitted Varshni relation.

In Figure 6a–c, results of spectral current responsivity (R_I_) measurements of considered photodetectors are shown for two reverse bias values each. Long-wavelength absorption edge λ_c_ was determined to be ~4.7 μm for all analyzed photodiodes. The cause of the slight differences in λ_c_ originates mainly from the variations in both the composition and thicknesses of InSb- and GaAs-like interfaces in the InAs/GaSb superlattice in the absorption region. The former were not taken into account during the simulation of 2θ/ω curves, in which binary interfaces were assumed. The latter are given in Table 1 in the section Fabrication of InAs/GaSb superlattice photodetectors. Dips in the spectra at wavelengths in the range of 3.3–3.5 μm in Figure 6a–c are caused by the absorption of radiation by water vapor present in the measurement chamber.

In Figure 6d, experimental values of bandgap energy E_g_ determined from R_I_ measurements at various temperatures for p-i-n photodiode are plotted. The data were fitted to Varshni relation (red line in Figure 6d), and the following parameters were obtained: E_g_0_ = 300 meV, α = 4.09 × 10^−4^ eV × K^−1^. The β = 270 K was assumed as it is most commonly used for InAs/GaSb type-II superlattices [30,31]. The fitting was performed for p-i-n photodiode only. For bariodes, R_I_ spectra were measured only at 2–3 different temperatures. The values of E_g_ of absorber region in p-i-n, p-B_p_bulk_-i-n, and p-B_p_SL_-i-n photodiodes at 210 K were 263 meV, 271 meV, and 273 meV, respectively. As was mentioned, the difference in E_g_ results from variation in thickness of InSb-like and GaAs-like interfaces in InAs/GaSb superlattice (Table 1).

The measured spectral current responsivity of p-i-n photodiode increased with reverse bias from ~1.2 A × W^−1^ for U = 0 V to ~1.65 A × W^−1^ for U = −0.9 V. It also had the highest values of R_I_, namely ~1.65–1.68 A × W^−1^ for wavelengths in the range from 3.55 μm to 4 μm. The photodiode with the AlSb barrier had the lowest R_I_, which was less than 0.04 A × W^−1^ in the entire measurement range. Two reverse biases were considered at 0 V and about −1 V. The presence of p-n junction in all described heterostructures should enable a passive operation of these photodetectors without bias. This is clear for the p-i-n diode, for which spectral current responsivity is nearly the same with and without bias. In principle, the addition of a barrier for electrons should not negatively impact carrier transport in the heterostructure and thus device operation. This is under the condition that the barrier layer will not introduce parasitic valence band offset between absorption and/or contact regions. Based on the curves shown in Figure 6b,c for U = 0 V, it is clear that an unwanted barrier for holes in the valence band is present. Both for p-B_p_bulk_-i-n and p-B_p_SL_-i-n bariodes, R_I_ was less than 5 mA × W^−1^. To overcome the potential barrier, an external bias was necessary.

Spectral current responsivity was measured for various biases from 0 V to 1 V. The values of the voltage result from the limitations of the measurement setup at the time. In the case of the p-i-n photodiode, the responsivity did not change with increasing voltage. For bariodes, both p-B_p_bulk_-i-n and p-B_p_SL_-i-n, R_I_ increased with reverse bias. Despite the improvement, the R_I_ for the photodetector with the AlSb barrier was very small, <40 mA × W^−1^. This indicates that the applied external electric field failed to completely overcome the parasitic barrier in the valence band and improve device operation. On the other hand, p-B_p_SL_-i-n bariode reached maximum R_I_ = 1.33 A × W^−1^, which corresponds to about 260 times improvement. This proves that AlSb/GaSb superlattice plays a much better role as an electron barrier than the bulk AlSb layer. Nevertheless, further optimization of the AlSb/GaSb structure is necessary to eliminate the parasitic valence band offset and improve device performance.

### 3.4. Specific Detectivity

Finally, the values of specific detectivity D* based on two semi-empirical approximations (D*_approx_.__1_, D*_approx_.__2_) and measured (D*_exp_) S_i_ were determined and juxtaposed. The calculations were performed for two bias voltages (0 V and ~−1 V), for λ = 4 μm, T = 210 K, and f = 10 kHz. In general, the D* for a bandwidth of 1 Hz can be defined as follows:

(2) $${D\;}^{*}=\frac{R_{I}\sqrt{A}}{\sqrt{S_{i}}},$$

where R_I_ is the spectral current responsivity, A is the area of the detector, and S_i_ is the noise power spectral density at a given frequency. The S_i_ can be further described as:

(3) $$S_{i}={\;S}_{i\_\mathrm{thermal}}+{\;S}_{i\_\mathrm{Schottky}}+{\;S}_{i\_\mathrm{lf}}+S_{i\_\mathrm{other}}=\frac{4k_{B}T}{R}+2\mathrm{qI}+\;\alpha \frac{I^{b}}{f}+S_{i\_\mathrm{other}},$$

where S_i_thermal_ is thermal noise PSD, S_i_Schottky_ is shot noise PSD, S_i_lf_ is low-frequency noise PSD, S_i_other_ is the sum of other (not included) noise power spectral densities, k_B_ is Boltzmann’s constant, T is temperature, R is dynamic resistance, q is the elementary charge, I is current, b is the power coefficient, which is usually assumed equal to 2, α is the noise coefficient, and f is frequency. In our case, the S_lf_ can be expressed as in Equation (3), assuming α = 2, due to the behavior of the data shown in Figure 5a. To most accurately determine the specific detectivity it is necessary to measure the noise PSD for a specific device. However, when this is not an option, it is possible to use semi-empirical approximations of S_i_. The first approximation (S_i_approx_.__1_) includes only thermal and shot noise components. It is calculated using I and R extracted from the measured current-voltage curves of a photodetector. The second approximation (S_i_approx_.__2_) includes thermal, shot, and low-frequency noise components. On top of I and R, it requires information about the noise coefficient α. In this approach, it is sufficient to measure α for a specific bias (not necessarily the voltage for which the detectivity will be determined), for example, −50 mV in this paper as shown in Figure 5c. Then, S_i_lf_ can be estimated using the expression given in Equation (3) for voltage bias, for which the S_i_(I) ~I^2^. Both approximations can be further simplified, depending on the bias of the photodiode. For unbiased photodetectors, in the absence of photocurrent due to background radiation, the specific detectivity will be limited only by thermal noise. The accuracy of these approximations depends on the interaction between the aforementioned noise components, which is related to the type of the photodetector, the quality of its structure, and processing. Furthermore, their usage may lead to both under- and overestimation of D*, especially under improper assumptions.

The first approximation (S_i_approx_.__1_) was used to estimate the specific detectivity D*_approx_.__1_ for unbiased and biased photodiodes in question. For U = 0 V the S_i_Schottky_ was about four, two, and one order of magnitude smaller than S_i_thermnal_ for p-i-n, p-B_p_bulk_-i-n, and p-B_p_SL_-i-n, respectively. The non-zero shot noise component originates from the photocurrent generated due to the background radiation. The following voltages, which were used during spectral current responsivity measurements, were used in the calculation of D* under bias: −0.9 V for p-i-n, −1 V for p-B_p_bulk_-i-n, and −1.12 V for p-B_p_SL_-i-n. In this case, the average of power spectral density magnitude of noise measured at 200 K and 225 K, for −1 V bias was taken as an approximation for the noise at T = 210 K. Based on the S_i_(T) function, it was determined that the character of changes of S_i_ in this temperature range allow for the use of such an approach. The results of the calculations under the following assumptions, T = 210 K, λ = 4 μm, and f = 10 kHz, are shown in Table 3. At zero bias, the highest values of D*_approx_.__1_, D*_approx_.__2_, and D*_exp_ were obtained for p-i-n photodiode and were equal to 1.61 × 10^10^ Jones, 1.61 × 10^10^ Jones, and 6.16 × 10^9^ Jones, respectively. They were about one to three orders of magnitude higher than for both bariodes, for which D*_approx_.__1_ = 1.43–1.52 × 10^8^ Jones, D*_approx_.__2_ = 7.05 × 10^7^–1.52 × 10^8^ Jones, and D*_exp_ = 1.04–1.25 × 10^8^ Jones. The main reason behind lower values was negligible current responsivity of bariodes in photovoltaic operation mode, which canceled out any performance improvement due to the decrease in noise. The p-B_p_SL_-i-n device outperformed p-i-n by about one order of magnitude in terms of S_i_, while p-B_p_bulk_-i-n by about two orders of magnitude. On the other hand, p-i-n achieved between 250 and 280 times larger R_I_ than other photodetectors.

The values of specific detectivity calculated using the first approximation decreased with a bias for simple photodiode mainly due to larger noise and increased a little for bariodes. For p-i-n photodiode, D*_approx_.__1_ decreased one order of magnitude with larger bias due to larger S_i_ to a value of 1.63 × 10^9^ Jones. At the same time, D*_approx_.__1_ increased slightly for p-B_p_bulk_-i-n to 2.44 × 10^8^ Jones and about an order of magnitude for p-B_p_SL_-i-n to 1.76 × 10^9^ Jones. The former is due to a similar increase in noise (14 times) and a decrease in R_I_ (4.64 times). On the other hand, S_i_ increased 681 times while R_I_ 300 times for the latter (D* is proportional to 1/√S_i_). The values of S_i_approx_.__2_ were from one to four orders of magnitude larger than those of S_i_approx_.__1_. Consequently, the values of D*_approx_.__2_ decreased for all photodetectors, as compared to D*_approx_.__1_, due to a large contribution of low-frequency noise estimated using semi-empirical approximation.

At zero bias, the values of S_i_approx_.__1_ were close to the experimental ones, especially so for p-i-n photodiode. As a result, D* behaved in the same way, which validates the use of this approximation in such conditions. On the other hand, at higher biases, the following was observed: S_i_approx_.__1_ << S_i_exp_ and D* _approx_.__1_ >> D* _exp_. This suggests that using only Johnson and shot noise components are not sufficient for proper approximation of the detectivity. This approach underestimated S_i_ for larger biases by two to four orders of magnitude, which proves that assumed noise components are not dominant for these structures and in these operating conditions. For p-i-n photodiode, D*_exp_ decreased more than an order of magnitude to 1.1 × 10^8^ Jones as compared to approximated value. A much smaller change was observed for the p-B_p_SL_-i-n photodetector (D*_exp_ = 1.04 × 10^8^ Jones). On the other hand, the p-B_p_bulk_-i-n device exhibited a decrease of two orders of magnitude to 1.3 × 10^6^ Jones. As a result, it had the worst performance even though it had the best noise characteristics.

The second approximation yielded values of S_i_approx_.__2_ for p-i-n and p-B_p_SL_-i-n, which were comparable to those estimated using the first approximation and the experimental ones. For p-B_p_bulk_-i-n the S_i_approx_.__2_ was close to the S_i_approx_.__1_, and both of them were a little larger (~2.75 times) than S_i_exp_. This difference originates from the assumption of the relation Si(I) ~I^2^, which does not hold for this bariode for small currents (Figure 5b). These results are expected since the 1/f noise is a non-equilibrium component, which vanishes as the current approaches zero. Consequently, the values of D*_approx_.__2_ were also similar to D*_approx_.__1_ and D*_exp_. At higher bias, the values of S_i_approx_.__2_ were overestimated by one and two orders of magnitude for p-i-n and p-B_p_SL_-i-n photodiodes, respectively. This suggests that the relation of S_i_(I) ~I^2^ does not hold for larger dark currents. This can be further confirmed by the data in Figure 5b, which shows this deviation for I > 4 × 10^−5^ A for p-i-n photodiode and I > 1 × 10^−5^ A for p-B_p_SL_-i-n bariode. At this point, the data for S_i_(I) for large currents were fitted to a power function without the assumption of b = 2 in Equation (3). The following values α and b were obtained: for p-i-n photodiode α = 5 × 10^−12^, b = 1.195; for p-B_p_SL_-i-n bariode α = 2.5 × 10^−12^, b = 1.195. These values were used to recalculate the S_i_ using the second approximation. The following results were obtained: for p-i-n S_i_approx_.__2_ = 2.82 × 10^−19^ (A^2^/Hz); for p-B_p_SL_-i-n S_i_approx_.__2_ = 6.29 × 10^−20^ (A^2^/Hz). Both values are very close to the experimental ones for these photodetectors. This proves that this approximation approach is correct given sufficient information about α and/or S_i_(I) relation. On the other hand, the value of S_i_approx_.__2_ was three orders of magnitude smaller than S_i_exp_ for p-B_p_SL_-i-n. This indicates that there are components of noise present in this detector other than thermal, shot, and low-frequency. Figure 5a,b show that the assumption of b = 2 holds for a wide range of currents, which proves that the proposed second approximation method can be useful in the determination of specific detectivity for many photodetectors.

As it was shown, the omission of the low-frequency noise during the calculation of the specific detectivity is unjustified without additional knowledge about its magnitude. Nevertheless, it is a common practice [32,33,34,35,36,37], even though the detector is voltage-biased. This approach can lead to a significant overestimation of detectivity in the detection of slowly varying IR sources.

## 4. Conclusions

In this paper, electrical, noise, and optical characteristics of p-i-n, p-B_p_bulk_-i-n, and p-B_p_SL_-i-n photodiodes were presented. The main subject was focused on device operation in HOT conditions. Both dark current and noise analysis showed that p-B_p_bulk_-i-n bariode had the best performance in these areas whereas p-i-n photodiode was the worst and p-B_p_SL_-i-n placed in-between. Optical characterization showed that at zero bias, p-i-n photodiode had the highest spectral current responsivity, while both bariodes had much lower values of R_I_. With higher bias R_I_ for p-i-n was still the highest, however, a larger increase in R_I_ was observed for p-B_p_SL_-i-n. The highest specific detectivity was achieved for p-i-n detector in photovoltaic operation mode. Both p-i-n and p-B_p_SL_-i-n photodiodes had almost the same values of D* at ~−1 V reverse bias. Obtained results indicate that at zero bias, both proposed electron barriers introduced parasitic valence band offsets, which effectively blocked the generated photocurrent. As such, the design of the bariodes could not be fully utilized. This suggests that with better optimization of electron barrier, a further improvement in the performance of bariode with AlSb/GaSb barrier could be achieved. This would be especially important at smaller bias, at which the gain from better noise performance in bariodes is more pronounced.

The specific detectivity of the photodiodes in question was determined using two semi-empirical approximations and empirical data. A simple method of incorporating the low-frequency noise contribution into the detectivity calculation, without time-consuming measurements, has been proposed. This approach is valid as long as the S_i_(I) ~I^2^ relation used in this approximation holds. It was shown that neglecting the low-frequency noise component can lead to a significant overestimation of detectivity.

## Figures and Tables

**Table 1:** The summary of structural details and lattice mismatch for photodetector heterostructures obtained from HRXRD characterization.

Structure	p-i-n	p-Bp_bulk-i-n	p-Bp_SL-i-n
Absorber			
GaAs IF thickness (ML/Å)	0.5/1.5	0.5/1.4	0.5/1.4
InAs layer thickness (ML/Å)	10.0/30.3	9.9/30	10.2/31
InSb IF thickness (ML/Å)	1.5/4.8	1.5/5.0	1.5/5.0
GaSb layer thickness (ML/Å)	10.1/30.7	9.8/30	9.9/30.1
(Δa/a)_⊥_ (ppm)	−920	−280	−280
electron barrier			
type	−	bulk AlSb	AlSb/GaSb SL
AlSb layer thickness (ML/Å)	−	−/500	4.4/13.6
GaSb layer thickness (ML/Å)	−	−	7.6/23.1
(Δa/a)_⊥_ (ppm)	−	11,800	4950

**Table 2:** Activation energies and corresponding temperature ranges for p-i-n, p-B_p_bulk_-i-n, and p-B_p_SL_-i-n photodiodes.

Photodetector	ΔT_1_	E_a1_ (meV)	ΔT_2_	E_a2_ (meV)	ΔT_3_	E_a3_ (meV)
p-i-n	>244 K	111	100–244 K	2.5	<100 K	1.4
p-B_p_bulk_-i-n	>240 K	220	112–240 K	2.6	<112 K	9.3
p-B_p_SL_-i-n	>260 K	132	104–260 K	9.2	<104 K	6.8

**Table 3:** The values of specific detectivity (D*) calculated for given biases, at λ = 4 μm, at T = 210 K, at f = 10 kHz.

Photodiode	U (V)	S_i_approx_.__1_ (A^2^/Hz)	S_i_approx_.__2_ (A^2^/Hz)	S_i_exp_ (A^2^/Hz)	D* _approx_.__1_ (Jones)	D* _approx_.__2_ (Jones)	D*_exp_ (Jones)
p-i-n	0	3.45 × 10^−24^	3.45 × 10^−24^	3.35 × 10^−24^	1.61 × 10^10^	1.61 × 10^10^	6.16 × 10^9^
	−0.9	6.55 × 10^−22^	3.89 × 10^−18^	1.45 × 10^−19^	1.63 × 10^9^	2.11 × 10^7^	1.1 × 10^8^
p-B_p_bulk_-i-n	0	9.8 × 10^−26^	1.11 × 10^−25^	3.59 × 10^−26^	1.43 × 10^8^	1.35 × 10^8^	2.4 × 10^8^
	−1	1.37 × 10^−24^	1.63 × 10^−23^	4.97 × 10^−20^	2.44 × 10^8^	7.05 × 10^7^	1.3 × 10^6^
p-B_p_SL_-i-n	0	4.87 × 10^−25^	4.88 × 10^−25^	7.28 × 10^−25^	1.52 × 10^8^	1.52 × 10^8^	1.25 × 10^8^
	−1.12	3.27 × 10^−22^	2.27 × 10^−18^	9.34 × 10^−20^	1.76 × 10^9^	2.12 × 10^7^	1.04 × 10^8^

## Data Availability

Data sharing does not apply to this article.
